# Analysis of Single Circulating Tumor Cells in Renal Cell Carcinoma Reveals Phenotypic Heterogeneity and Genomic Alterations Related to Progression

**DOI:** 10.3390/ijms21041475

**Published:** 2020-02-21

**Authors:** Vera Cappelletti, Elena Verzoni, Raffaele Ratta, Marta Vismara, Marco Silvestri, Rosanna Montone, Patrizia Miodini, Carolina Reduzzi, Melanie Claps, Pierangela Sepe, Maria Grazia Daidone, Giuseppe Procopio

**Affiliations:** 1 Biomarker Unit, Department of Applied Research and Technological Development, Fondazione IRCCS Istituto Nazionale Tumori di Milano, 20133 Milano, Italy; vera.cappelletti@istitutotumori.mi.it (V.C.); Marta.Vismara@istitutotumori.mi.it (M.V.); marco.silvestri@istitutotumori.mi.it (M.S.); patrizia.miodini@istitutotumori.mi.it (P.M.); carolina.reduzzi@istitutotumori.mi.it (C.R.); mariagrazia.daidone@istitutotumori.mi.it (M.G.D.); 2 Department of Medical Oncology, Fondazione IRCCS Istituto Nazionale Tumori, 20133 Milano, Italy; raffaele.ratta@gmail.com (R.R.); melanie.claps@istitutotumori.mi.it (M.C.); pierangela.sepe@istitutotumori.mi.it (P.S.); giuseppe.procopio@istitutotumori.mi.it (G.P.); 3 Clinical Trial Center, Fondazione IRCCS Istituto Nazionale Tumori, 20133 Milano, Italy; rosanna.montone@istitutotumori.mi.it

**Keywords:** renal cell cancer, circulating tumor cells, heterogeneity, cancer evolution, copy number alterations, single-cell analysis

## Abstract

Circulating tumor cells (CTCs) are promising biomarkers for prognosis, therapeutic response prediction, and treatment monitoring in cancer patients. Despite its epithelial origin, renal cell carcinoma (RCC) shows low expression of epithelial markers hindering CTC-enrichment approaches exploiting epithelial cell surface proteins. In 21 blood samples serially collected from 10 patients with metastatic RCC entering the TARIBO trial, we overcame this limitation using the marker-independent Parsortix™ approach for CTC-enrichment coupled with positive and negative selection with the DEPArray™ with single cell recovery and analysis for copy number alterations (CNA) by next generation sequencing NGS. Two CTC subpopulations were identified: epithelial CTC (eCTC) and non-conventional CTC (ncCTC) lacking epithelial and leukocyte markers. With a threshold ≥1CTC/10 mL of blood, the positivity rates were 28% for eCTC, 62% for ncCTCs, and 71% considering both CTC types. In two patients with detectable eCTCs at baseline, progression free survival was less than 5 months. In an index case, hierarchical structure by translational oncology (TRONCO) identified three clones among 14 CTCs collected at progression and at baseline, each containing cells with a 9p21.3loss, a well-known metastasis driving subclonal alteration. CTCs detection in RCC can be increased by marker-independent approaches, and CTC molecular characterization can allow detection of subclonal events possibly related to tumor progression.

## 1. Introduction

About 30% of patients with renal cell carcinoma (RCC) present metastatic disease at diagnosis [1]. The prognosis of metastatic RCC (mRCC) is poor, with an overall 5-year survival rate of lower than 20% [1]. In the past decade, targeted therapies (TTs) have permitted prolonged progression free survival (PFS) and overall survival (OS) in this setting. Among TTs, sunitinib [2] and pazopanib [3], approved as first-line treatment options for mRCC patients, showed comparable efficacy in terms of PFS and OS [4]. However, the role of nephrectomy remained controversial in metastatic diseases. Two randomized phase three trials showing a significant improvement in OS for patients receiving cytoreductive nephrectomy (CN) followed by IFN-α compared to IFN-α alone support the role of CN as a part of treatment for mRCC patients [5,6,7]. More recently, the CARMENA trial showed the non-inferiority of a treatment with sunitinib alone over nephrectomy followed by sunitinib in patients with intermediate-risk or poor-risk metastatic renal cell carcinoma [8]. Thus, the identification of patients that may really benefit from a combination of CN and tyrosin kinase inhibitors (TKI) represents a challenge for clinicians. In this regard, the explorative objective of the TARIBO trial was the development of non-invasive biomarkers that predictive clinical outcome, thus helping treatment choice and patients’ selection.

Circulating blood biomarkers (CBBs), including circulating tumor DNA (ctDNA) and circulating tumor cells (CTCs), represent promising biomarkers for predicting prognosis and monitoring response/resistance to treatment in cancer. In addition, CBBs may reflect the intratumor heterogeneity and offer a picture of the tumor genetic landscape, helping clinical management in the absence of available metastatic tissue samples [9,10,11,12,13,14,15].

Since intratumoral heterogeneity limits the validity of molecular information obtained from single biopsies [16], we hypothesized that CTC analysis at single-cell level helps uncovering spatial heterogeneity, offers a hint on temporal heterogeneity (through longitudinal blood sample collection), and contributes to identification of clones driving tumor progression.

Presently, only enumeration of CTC using the CellSearch™ (Menarini Silicon Biosystems) has received approval by the FDA for use in treatment monitoring in metastatic prostate breast and colon cancer. The CellSearch™, which exploits antibodies against epithelial cell adhesion molecule (EpCAM) for CTC enrichments and checks expression of cytokeratins (CKs) for CTC identification, is not the optimal tool for evaluating CTCs in every tumor type. Indeed, in patients with RCC, a tumor that, despite its epithelial origin, is characterized at tissue level by low EpCAM and low CK expression [17], the use of CellSearch™ did not provide relevant results. Not surprisingly, a study including 11 patients with mRCC reported the presence of at least 1 CTC/7.5 mL of blood only in 16% of patients [18] and suggested the presence of distinct CTC subpopulations not matching the classic CellSearch™ criteria. Therefore, improving CTC detection in mRCC by developing alternative CTC detection approaches will help unlock their informative content and offer important hints in treatments planning of this disease.

We tested our hypothesis on the clinical relevance of CTCs in a subset of patients from the TARIBO study [19], an Italian multicenter phase three study comparing sunitinib or pazopanib vs. CN followed by sunitinib or pazopanib for patients with mRCC who have not received surgery and prior systemic treatment for metastatic disease. In blood samples collected from patients enrolled in the TARIBO trial, we ran a head-to-head comparison of two different CTC detection methods: the AdnaTest that employs a positive selection for CTC-enrichment based on antibody-mediated recognition of surface markers followed by multiplex RT-PCR for epithelial or tumor-associated transcripts, and a new method developed in our laboratory that performs an unbiased CTC enrichment and allows enumeration and isolation of single CTC for downstream molecular analyses [20]. Despite the low number of patients (due to discontinuation of the TARIBO trial), we observed a strong heterogeneity in CTCs both at phenotypic and at molecular level, identified a new subpopulation of non-conventional CTCs lacking epithelial markers, and provided preliminary evidence on the prognostic role of the classic epithelial CTCs. In a case study, genomic profiling of 14 CTCs isolated from a patient experiencing clinically documented disease progression revealed the presence hallmark genomic losses at chromosome 9p and 14q, which represent known genomic drivers of metastasis in RCC [21].

## 2. Results

### 2.1. Blood Collection for CTC Studies

We collected blood samples from ten patients recruited at our Institution (four in the CN arm and six in the treatment-only arm). Baseline (BL) blood samples were available for nine out of 10 patients, samples collected during treatment (DT) were available for seven patients, and blood at progression was available for three patients only, whereas postsurgical samples were available in all four patients who underwent CN. CTC determination was performed on all available samples without knowledge of clinical data by using two methods as described in Material and Methods. Only for two cases (patients K016 and K017) were blood draws not evaluable when processed with the DEPArray™ due to a technical failure. Table 1 reports CTC results and clinical outcomes for each single patient.

### 2.2. DEPArray CTC Counts

Globally, considering all the 21 blood samples successfully processed with the DEPArray™, we were able to identify 15 CTCs expressing epithelial markers (eCTCs) corresponding to the classical CTC definition and 22 CTCs lacking both epithelial and leukocyte markers. Those latter CTCs that did not meet the classical CTC definition were characterized by aberrant genomes and were considered as non-conventional CTC (ncCTCs).

When considering as positivity threshold ≥1 CTC/10 mL of blood, the positivity rate in our blood samples was 6/21 (28%) for eCTC, increased to 13/21 (62%) for ncCTCs, and was 15/21 (71%) when considering both CTC types (CTC tot). Both for eCTC and ncCTC, median values did not differ between BL (including pre-surgical samples) and DT samples (zero vs. zero and one vs. one for eCTC and ncCTC, respectively). Only in one of the three patients for whom blood draws at progression were available was a clear increase in the number of eCTC and ncCTCs observed (one eCTC/10 mL vs. nine eCTC/10 mL and zero ncCTC/10 mL vs. three ncCTC/10 mL).

### 2.3. CTC Status by AdnaTest

Overall, considering 23 blood samples available for the AdnaTest, the CTC status was defined as positive in 8/23 samples (35%) and was not influenced by the blood drawing time (Fisher exact test *p* = 0.538). The results on CTC status obtained with the AdnaTest were discordant with the CTC status defined with the DEPArray™ method when considering both eCTC (Cohen’s kappa = −0.27) and ncCTCs (Cohen’s kappa = −0.17).

### 2.4. CTC Status Clinical Correlates

After 6 months of treatment, four patients were rated as progressive disease (PD), three as stable disease (SD), and one patient showed a partial response (PR). For this last patient, blood draws were negative for all types of CTC determinations at all available times, and the patient was still disease-free after 18 months. On the contrary, in the patients with SD or PD, no clear associations were observed with either baseline CTCs or with CTCs during the treatment.

Since the number of patients with evaluable follow-up time was very limited, we do not report here a formal statistical analysis to explore the associations between the baseline CTC results obtained with the various methods and the OS and the PFS. However, we noticed that the only two patients scored as eCTC-positive at BL were characterized by a less than 5 months PFS with respect to a median PFS superior to 13 months in patients negative for eCTC at BL.

### 2.5. Molecular Characterization of Single CTCs

**Figure 1:**
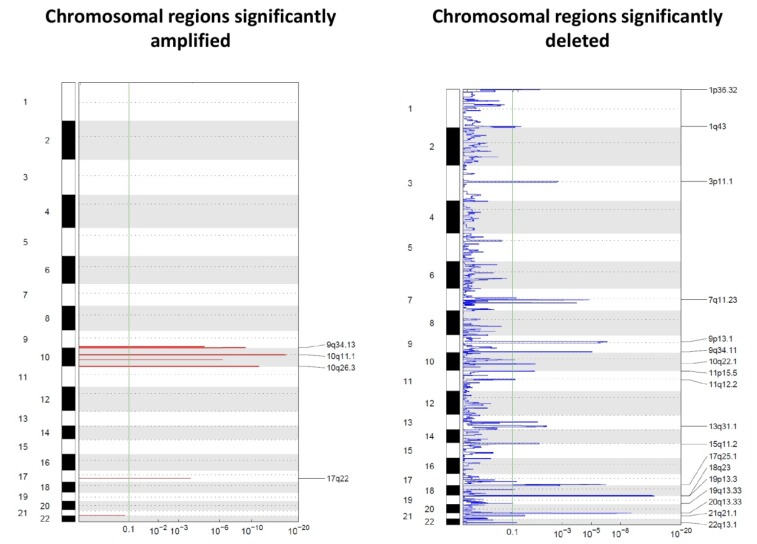
Genomic Identification of Significant Targets in Cancer (GISTIC) amplification (left) and deletion (right) plots on single CTCs. The genome is oriented vertically from top to bottom, and the GISTIC *q*-values at each locus are plotted from left to right on a log scale. The green line represents the significance threshold (*q*-value = 0.1).

The molecular characterization of single cells recovered with the DEPArray™ provided additional information by identifying chromosomal regions affected by copy number alterations. Only 32 CTCs (13 eCTCs and 19 ncCTCs) for which at least 500,000 reads were available by whole genome sequencing (WGS) were used for this analysis (Figure 1). Data are reported in Figure 1 as Genomic Identification of Significant Targets in Cancer (GISTIC) plots. GISTIC is a tool that evaluates the frequency and the amplitude of segmented copy number values. We noticed that regions that have been reported to be frequently affected by copy number gains and losses in RCC were also consistently affected by genomic alterations in our cells, thus supporting the technical reliability of our data and the possible tissue surrogacy by CTCs [21,22].

It is immediately clear that deletions prevailed over amplifications. None of the regions affected by amplifications or losses included canonical suppressor genes often mutated in RCC, such as *VHL*, *PBRM1 SETD2*, or *BAP1*. However, losses involved regions on chromosomal arms such as 1p, 9p, and 20q, which have already been described in the literature as frequently undergoing losses during tumor evolution.

On the front of genomic gains, the 10q26.3 region was among the regions showing the most frequent gains (in 62% of the analyzed CTCs). Such a region contains around 50 genes, including *ADAM8*, a membrane metalloproteinase involved in the development of RCC described to be associated with shorter OS and with distant metastases development [23].

**Figure 2:**
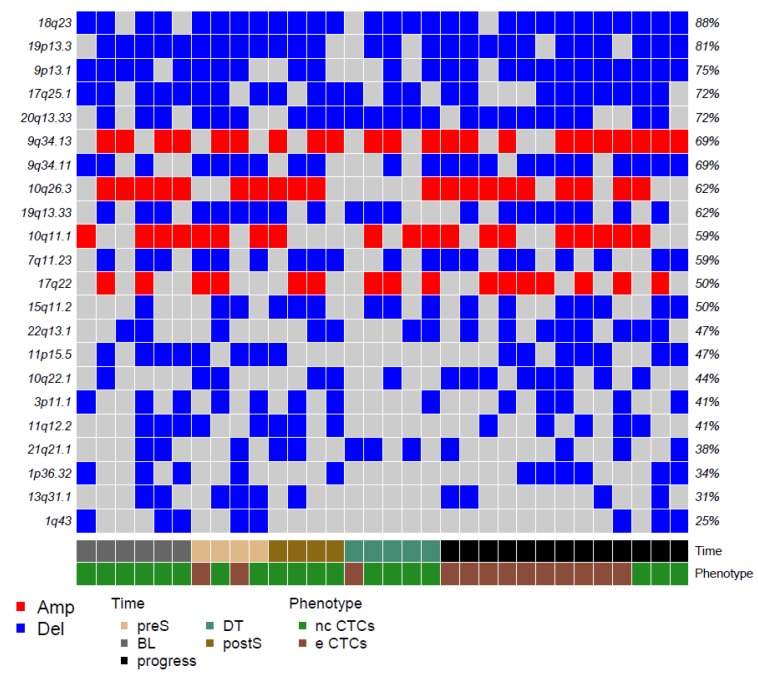
Matrix reporting the top genomic gains (red) and losses (blue) in our CTCs. Color codes refer to blood collection timing and CTC phenotype.

Among regions affected by genomic losses, the chromosomal region 3p11.1, which includes *EPHA3*, appears as particularly interesting for RCC. *EPHA3* codes for a member of the ephrin receptor tyrosine kinase family, whose loss has been described as linked with progression in clear cell RCC [24]. Figure 2 reports the 22 top alterations detected with GISTIC.

**Figure 3:**
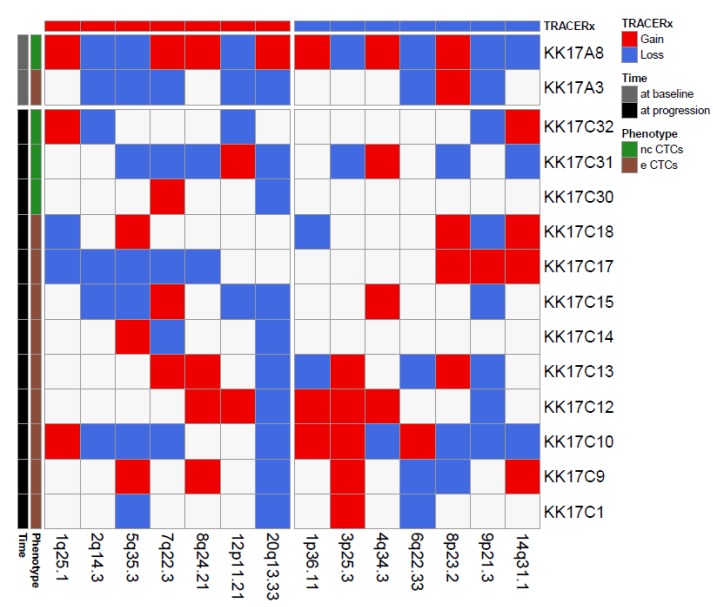
Matrix reporting genomic gains (red) and losses (blue) commonly observed in renal cell cancer for 14 CTCs isolated from patient K017. Color codes refer to blood collection timing and CTC phenotype.

We next focused on a single case, patient K017, a 68-year-old women diagnosed with a clear cell RCC with lung and brain metastases showing progressive disease 5 months after CN and sunitinib treatment. Since, for this patient, we successfully isolated and profiled 14 CTCs, i.e., one eCTC and one ncCTC in a blood sample collected pre-surgery, nine eCTCs and three ncCTCs in blood obtained at progression, we could use our CNA data to investigate the presence of single cells deriving from clones enriched in genomic alterations that have been described in the literature as metastatic drivers of CNA [21]. In particular, by comparing our data with chromosome regions frequently involved in losses at tissue level in the TRACERx study (Figure 3), we noticed that the region 9p21.3 was the most frequently lost among the CTCs from this patient (8/14 CTCs independently from the specific phenotype).

Loss of chromosome 9p21.3 was reported as a selected event in metastasis in the TRACERx landmark study, where it was shown to be significantly enriched across three distinct cohorts (TRACERx Renal; Hospital Universitario Cruces, HUC; Memorial Sloane Kettering, MSK). Moreover, loss at this region, which contains two members of the cyclin-dependent kinase family (p16/p14ARF and p15^INK4b^), was also associated with reduced OS in the HUC and the TRACERx cohorts (no OS data were available for the MSK cohort). Similar to what was reported in the literature for OS, our K017 patient bearing the loss at 9p21.3 in the majority of its CTCs had a shorter PFS (4 months, i.e., 6 months earlier than the median PFS of our case series). 

**Figure 4:**
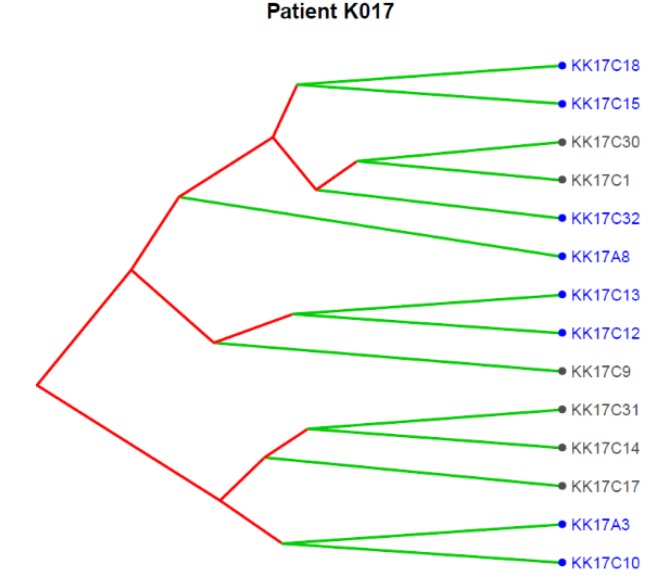
Translational oncology (TRONCO) was used to order 14 single CTCs isolated from patient K017 into a clonal hierarchy. The figure represents phylogenies of single CTCs grouped into three distinct clones by considering genomic regions altered in at least 50% of all CTCs. Loss at 9p21.3 (indicated by blue color) was observed across all the three clones.

Figure 4 reports the clonal structure of the 14 CTCs isolated in patient K017 as inferred from CNA data at single-cell level. Three major clones were identified, and we noticed that the 9p21.3, as expected for a subclonal event, was present in some CTCs in each of the three clones, supporting its role as an alteration strongly selected at progression.

## 3. Discussion

Until June 2018, when data of the CARMENA trial were presented, no prospective data were available regarding the role of CN in mRCC. In this trial, mRCC patients were randomized to receive CN and then sunitinib or sunitinib alone. The results in the sunitinib-alone group were non-inferior to those in the nephrectomy-sunitinib group with regard to overall survival, and no significant differences in response rate or PFS were observed [8]. Due to availability of these results and to the slowness in the accrual, the enrollment in the TARIBO trial was prematurely closed. However, despite the limited patient accrual that prevented us from optimally addressing the clinical objectives of the trial, the exploratory objectives that dealt with the relevance of CTC isolated at baseline in predicting clinical outcome and with longitudinal molecular characterization of CTCs to identify genomic alterations fostering progression could be completed.

Therefore, we are reporting here the translational results only, referring to the clinical relevance of CTC detection, discussing methodological issues, and presenting, for the first time in this tumor type, molecular CTC studies at a single cell level. Indeed, despite the fact that only ten patients were included in CTC studies, the collected data are innovative and offer a proof of concept on the relevance of our CTC detection approach thanks to the successful isolation of different CTC subpopulations and to the molecular characterization of 14 individual CTCs from a single patient that were collected across baseline and progression.

CTCs have been studied in different cancer types, including urologic tumors, proving to be a non-invasive and feasible approach with different clinical implication, including prediction of recurrence and response to treatment, disease staging, and prognostication. Antonarakis et al. demonstrated the predictive role of CTCs status combined with ARV7 detection in patients affected by metastatic castration resistant prostate cancer [25,26]. Moreover, in patients undergoing radical cystectomy for bladder cancer, CTCs showed to be a useful prognostic biomarker [27]. However, compared to other genitourinary tumors, less is known regarding CTCs in kidney cancer, mainly due to the low expression of EpCAM, the epithelial cell surface protein commonly used for CTC assays. 

In our 10 mRCC patients, CTC status positivity rates of samples analyzed by the AdnaTest were low (35%) despite the fact that patients presented metastatic diseases. This low detection rate supports the need to use unbiased CTCs collection methods to be able to capture the entire CTC population (i.e., conventional CTCs expressing epithelial markers and non-conventional CTC lacking epithelial markers). Indeed, thanks to technical improvements made possible by the Parsortix technology coupled with the use of DEPArray™, which offered the possibility to recover single CTCs or small CTCs pools devoid of leukocyte contamination, we could obtain an improved CTC identification with a global CTC positivity rate (eCTC+ncCTC) of 76%. Nonetheless, the median number of CTCs remained low (median = 1, range 0–12, *n* = 21), but it was noteworthy that, in samples collected at baseline/presurgery, despite the extremely low CTC counts, the presence of even one single eCTC was associated with a shorter PFS.

CTCs from patients with RCC are particularly heterogeneous both at phenotypic and genotypic levels, possibly due to the involvement of chromosomal remodeling genes in RCC etiology [22]. Moreover, most renal tumors are characterized by low expression of cytokeratins and EpCAM. As a consequence, the classic FDA-approved CellSearch^®^ approach, which detects only cells expressing epithelial markers, has been rarely used in this neoplasia [18,28]. Indeed, other groups similarly attempted what we attempted—To use marker-independent CTC capture approaches spanning from density gradient centrifugation for negative depletion of leukocytes and filtration-based methods [29,30]. In all such studies, CTC identification was based on the expression of specific biomarkers evaluated either by PCR or by immunofluorescence or was simply based on morphology. New cell surface markers have been recently used, including membrane CAIX and CD147, in conjunction with the NanoVelcro platform, obtaining a significant increase in CTC positivity rates [31]. Among new methods that have been used to overcome limited sensitivity of EpCAM based methods, there are marker independent methods such as subtraction-enrichment immunostaining fluorescent in situ hybridization (SE-iFISH), which identifies CTCs based on chromosome 8 aneuploidy and characterizes them further based on the expression of a set of other biomarkers (*CK18*, *EPCAM*, *PDL1*, *VIM*) after having enriched the blood samples by subtraction of CD45-positive cells [32].

We showed here that using a marker-independent approach for CTC enrichment was instrumental for increasing CTC detection and for being able to separately identify the subpopulations by immunostaining. We obtained similar results using the same method in the context of patients with advanced biliary tract cancer, similar to the trend observed in RCC, where baseline eCTCs were predicting clinical outcome [33]. Indeed, patients where at least one eCTC/10 mL of blood was detectable prior to treatment start were characterized by a significantly shorter OS than those with undetectable eCTCs, supporting their prognostic relevance, whereas—Different from what was observed in RCC—In biliary tract cancer patients, changes in the numbers of ncCTCs were able to anticipate treatment response.

Importantly, beyond proving the malignant nature of CTCs, results from CNA were also informative for identification of genomic regions frequently altered in mRCC patients and that might be involved in metastatic progression. Tumor heterogeneity and tumor cell evolution over time still represent major hurdles in the treatment of cancer [13]. As a result, often genetic alterations that bear metastatic potential are already present at the subclonal level in the primary tumor and may be missed when sequencing single samples from the tissue. CTCs released in the blood stream by both the primary tumor and metastases can provide a snapshot on clones responsible for tumor dissemination and progression. However, minor clones may be difficult to detect in CTC-enriched fractions, and only single cell analysis can enable detection of such clones. We described among our patients an index case that supports the importance of studying single CTCs for understanding tumor evolution and put it into the context of state-of-art studies in RCC [21].

The TRACERx renal study showed that cellular clones bearing chromosomes 9p21.3 and 14q31.1 loss are frequently enriched in metastatic disease and act as drivers of the dissemination process. In fact, in blood samples from patient K017, prior to CN, two CTCs representing distinct clones were found, one harboring 9p21.3 and 14q loss and the other with only the 9p21.3. At the time of disease progression, when the primary tumor had already been removed by CN, and thus CTCs could only be released from the metastatic site, almost 65% of isolated CTCs presented those alterations.

Indeed, as observed by Turajlic and colleagues [21], loss at 9p21.3, despite being a potentially useful biomarker for RCC patients, can be missed when analyzing a single region of the primary tumor since it is a subclonal event. 

Therefore, beside eCTC enumeration, molecular characterization of both eCTC and ncCTC provide clinically valid information that is not readily available at the tissue level. However, the low number of detected CTC still represents the major limitation. Finally, single cell analysis can inform clonal evolution and phylogenesis, but again, we have here CTC numbers too low to allow speculation on the disease evolution.

## 4. Materials and Methods

### 4.1. Patients

Eligible patients for the TARIBO study were adults with age between ≥ 18 years and ≤ 75 years affected by a metastatic predominantly clear cell RCC. Up to three different metastatic sites and at least three metastatic lesions had to be documented. Oligometastatic disease suitable for metastasectomy (less than 3 lesions confined at one organ site) was excluded. Patients were required to be classified as good or intermediate Memorial Sloan Kettering Cancer center (MSKCC) score or Heng risk score and to have Eastern Cooperative Oncology Group (ECOG) performance status of 0 and 1, absence of brain and liver metastases, and adequate bone marrow, liver, renal, and pancreatic function. The tumor had to be considered suitable to nephrectomy by a urologist, and patients had to be considered eligible for treatment with sunitinib or pazopanib before enrollment. Patients with respectable in situ primary tumors were considered eligible in the absence of symptoms that could be exclusively assigned to the primary tumor, such as flank pain and/or gross hematuria necessitating blood transfusion. Patients were excluded if they had diagnosis of bilateral RCC, non-clear cell histology, concomitant cardiac disorders, and if they had received prior therapies for advanced disease. Adjuvant treatment was admitted if stopped at least six months before entry into the study. All the patients provided written informed consent before undergoing any trial procedures.

### 4.2. Objectives

In this study, we addressed the translational exploratory objective of the TARIBO dealing with the relevance of CTCs in predicting the clinical outcome and in identifying molecular drivers of progression.

### 4.3. Blood Collection

All the patients provided written informed consent before undergoing any trial procedures, and the CTC study was approved by the Istituto Nazionale dei Tumori (INT) Institutional Review Board and Ethics Committee on 23 July 2015 with the identification code INT 75/15. The study involved the collection of blood samples for dynamic analyses of blood biomarkers. 

Blood samples were collected at baseline (BL), pre- and post-operatively (in patients undergoing CN), 24 weeks after randomization (DT), and at the time of PD, as shown in Figure S1. Samples of peripheral venous whole blood were drawn using a 21 G needle and collected in 4 mL K3EDTA or 6 mL K2EDTA BD Vacutainer tubes if intended to be processed with the AdnaTest (AdnaGen, AG, Langenhagen, Germany) or the Parsortix™ (Angle plc, Guildford, UK), respectively. In order to minimize the risk of contamination with epithelial skin cells during puncture, the first mL of blood was discarded. Fresh samples were stored at 4 °C in the dark and processed within 2 h.

### 4.4. Circulating Tumor Cell (CTCs) Assessment

#### 4.4.1. AdnaTEST for CTC Status Assessment

The AdnaTest ProstateCancerSelect kit (AdnaGen, AG, Langenhagen, Germany), which uses a positive CTC-selection method, was used for CTC enrichment. Briefly, 5 mL of whole blood were incubated with 100 μL of magnetic beads and coated with antibodies against the epithelial and the tumor-associated antigens EpCAM and ErbB2 on a tube rotator for 25 min at room temperature. Cell–bead complexes were captured using the AdnaMag-L magnetic particle concentrator and washed in Dulbecco’s phosphate-buffered saline (DPBS). Cell lysates were stored at −20 °C, and downstream molecular analyses were performed within 2 weeks.

For CTC identification, the expression of *EPCAM, MUC1*, and *ERBB2* was assessed by semiquantitative multiplex-PCR according to the manufacturer’s instructions using the AdnaTest BreastCancerDetect kit (AdnaGEN). Briefly, mRNA was isolated using Dynabeads^®^ Oligo(dT)25 and retrotranscribed in a final volume of 40 µl. The multiplex-PCR was performed using the BreastCancerDetect kit. PCR products were analyzed on the Agilent 2100 Bioanalyzer (Agilent Technologies, Santa Clara, CA, USA) using the DNA 1000 kit (Agilent Technologies). Samples with *ACTB* concentration ≥ 3.0 ng/µL were considered good quality. Samples were considered as CTC positive when at least one among *EPCAM, MUC1*, and *ERBB2* markers was higher than the previously defined cut off values, (≥0.40 ng/µL for *EPCAM*; ≥0.10 ng/µL for *MUC1*; and ≥0.20 ng/µL for *ERBB2* [34]. Samples with all marker concentrations under the cut off values were defined as CTC negative.

#### 4.4.2. CTC Identification and Recovery by DEPArray™ for Single Cell Studies

The protocol for CTC identification and recovery by DEPArray was previously described in detail [20].

Blood samples (10 mL) collected in K_2_EDTA tubes were subjected to CTC enrichment with Parsortix (Angle plc, Guildford, UK) within 1 h from blood draw [35]. Enriched cells were harvested according to manufacturer’s instructions and fixed for 20 min at room temperature (RT) with 2% paraformaldehyde. Fixed samples were stained immediately or within 24 h from enrichments. Mean Parsortix’s recovery rates (previously investigated using 5 different cell lines spiked in healthy donor blood at final concentrations ranging between 25–50 cells/10 mL) were 81% (range 75–90%, depending on the cell line) [20].

Fixed samples were fluorescently stained with phycoerythrin (PE)-labeled antibodies against epithelial markers EpCAM (clone HEA-125, Miltenyi Biotec, Bergisch Gladbach, Germany, working dilution 1:11 for 10 min at 4 °C), cytokeratins (pan cytokeratin clone C11, Abcam, San Francisco, CA, USA, and pan cytokeratin clone AE1/AE3, NSJ Bioreagents, San Diego, CA, USA, working dilution 1:10 for 10 min at RT) and EGFR (clone 423103, SantaCruz Biotechnology, Dallas, TX, USA, working dilution 1:11 for 10 min at 4 °C), and with allophycocyanin (APC)-labeled antibodies recognizing leukocytes and monocytes: CD45 (clone 5B1, Miltenyi Biotec, working dilution 1:11 for 10 min at 4 °C), CD14 (clone M5E2, BD Biosciences Pharmigen, San Diego, CA, USA, working dilution 1:20 for 10 min at 4 °C), and CD16 (clone 3G8, BD Biosciences Pharmigen, working dilution 1:20 for 10 min at 4 °C). Nuclei were stained with 1 µg/mL Hoechst 33342 (Sigma-Aldrich, Saint Louis, MI, USA) for 5 min at RT. The labeling procedure did not lead to significant loss of cells.

Labeled cells were analyzed using the DEPArray™ (Menarini Silicon Biosystems, Bologna, Italy) within 2 days from staining to visualize and recover single cells manually selected based on fluorescence labeling and morphology [36]. Selected single epithelial or double-negative (PE-ve/APC-ve) cells were recovered for downstream molecular analyses. CTC enrichments by Parsortix lasted about 3 h, fixation lasted 20 min, and the cell selection and recovery process with the DEAPrray lasted about 3–4 h depending on the number of cells recovered from the patient

### 4.5. Molecular Characterization of Isolated CTCs

Recovered single cells and pools of white blood cells (WBC) were subjected to whole genome amplification employing the Ampli1™ WGA kit (Silicon Biosystems). Amplified DNA quality was checked with the Ampli1™ QC kit (Silicon Biosystems), and a low-pass whole genome sequencing (lpWGS) to detect copy number aberrations (CNAs) was performed using the Ampli1™Low Pass kit (Silicon Biosystems) for barcoded libraries preparation, followed by sequencing with the IonTorrent Ion S5™ system (Thermo Fisher, Waltham, MA, USA), using the Ion530 chip according to the manufacturer’s instructions.

### 4.6. Bioinformatics Analyses

WGS sequences were aligned to the human reference genome (hg19) using tmap aligner tool on Torrent_Suite 5.4.0. CNAs were predicted by using control-FREEC 11.0 with the following settings: coefficientofVariation = 0.05, mateOrientation = 0, sex = XY or XX. control-FREEC produced different window size according to the sequencing depth in each sample. “Gain” and “loss” calls were filtered out when the Wilcoxon Rank-Sum Test, and Kolmogorov–Smirnov *p*-values were greater than 0.05.

Segmented copy number data of each sample were extracted starting from logRatio value and submitted to GISTIC 2.0 [37]. The software identifies regions and corresponding genes targeted by frequent and significant somatic copy number alteration. TRONCO (TRanslational ONCOlogy), a tool to infer progression models [38] and hierarchical clustering analysis, was used to identify the clonal hierarchy of K017 patient starting from genomic regions altered in at least 50% of CTCs.

### 4.7. Clinical Correlates

Progression-free survival (PFS) was calculated from date of enrollment to the date of detection of imaging-documented progression. Overall survival (OS) was calculated from the date of enrollment to the death date or censored at the date of last follow-up for living patients (median follow-up = 28 months, range = 9–48 months). 

## 5. Conclusions

In conclusion, as pointed out in the literature, CTC studies in kidney cancer patients are very limited due to the difficulty of tracing CTCs in the blood of patients, even when at advanced disease stages. Taking advantage of the TARIBO clinical trial, we reported here CTC status and CTC molecular features on a small number of patients with mRCC. Our data confirm the heterogeneity of CTCs in RCC [29] and suggest that detection of even one single eCTC prior to systemic treatment start predicts a short PFS.

However, despite having overcome selection biases due to low expression epithelial markers and having run in parallel CTC methods addressing bulk CTC at mRNA level and single cell CTC at DNA level characterizations, we could not prove the validity of our approach for treatment response monitoring and for OS prediction due to the limited number of patients. Nonetheless, the possibility to obtain reliable molecular data on the molecular features of single CTCs opens new perspectives, as demonstrated by results obtained with our index patient proving that single cell analysis of CTCs unlocks the presence of hidden clones with metastatic potential.

## Figures and Tables

**Table 1:** CTC and treatment response data in the TARIBO case series.

		CTC by DEPArray			AdnaTest	Clinical Outcomes				
Patient ID	Time	eCTC	ncCTC	CTC tot	CTC Status	Response	PD	PFS (mos) *	DEATH	OS (mos)
K009	preS	1	0	1	pos	PD	yes	4.14	yes	8.98
	postS	0	1	1	pos					
	DT	0	0	0	pos					
K012	preS	0	1	1	neg	SD	yes	13.22	yes	22.07
	postS	0	3	3	pos					
	DT	0	0	0	pos					
	progress	1	0	1	neg					
K016	preS	/	/	/	/	RP	no	28.22	no	28.22
	postS	0	0	0	neg					
	DT				neg					
K017	preS	2	2	4	neg	PD	yes	4.74	no	20.13
	postS				neg					
	progress	9	3	12	neg					
K008	BL	0	0	0	neg	SD	yes	36.32	no	48.06
	DT	1	2	3	neg					
K010	BL	0	2	2	neg	NA	NA		yes	3.39
K011	BL	0	1	1	pos	SD	yes	42.83	no	42.83
	DT	0	1	1	neg					
K013	BL	0	2	2	neg	NA	NA		yes	7.99
K014	BL	0	0	0	neg	PD	yes	5.56	no	8.75
	DT	0	1	1	neg					
K015	BL	0	2	2	pos	PD	yes	8.222	yes	26.84
	DT	1	1	2	neg					
	progress	0	0	0	pos					

* months. CTC and treatment response data in the TARIBO case series. Abbreviations: CTC, circulating tumor cells; eCTC, epithelial CTC; ncCTC, non-conventional CTC; PFS, progression free survival; OS, overall survival; preS, pre-surgery; postS, post-surgery; DT, 24 h after randomization; BL, baseline; PD, progression disease; SD, stable disease; NA, not available.

## Supplementary Materials

Supplementary materials can be found at https://www.mdpi.com/1422-0067/21/4/1475/s1.

## Abbreviations

CTC	Circulating tumor cells
RCC	Renal cell carcinoma
CNA	Copy number analysis
PFS	Progression-free survival
OS	Overall survival
CN	Cytoreductive nephrectomy
TKI	Tyrosin kinase inhibitor
CBBs	Circulating blood biomarkers
BL	BaseLine
DT	During treatment
