# Supplementary figures and images for: Analysis of Single Circulating Tumor Cells in Renal Cell Carcinoma Reveals Phenotypic Heterogeneity and Genomic Alterations Related to Progression
Source: Int J Mol Sci. 2020 Feb 21;21(4):1475. doi: 10.3390/ijms21041475 (PMC7073151; doi:10.3390/ijms21041475)

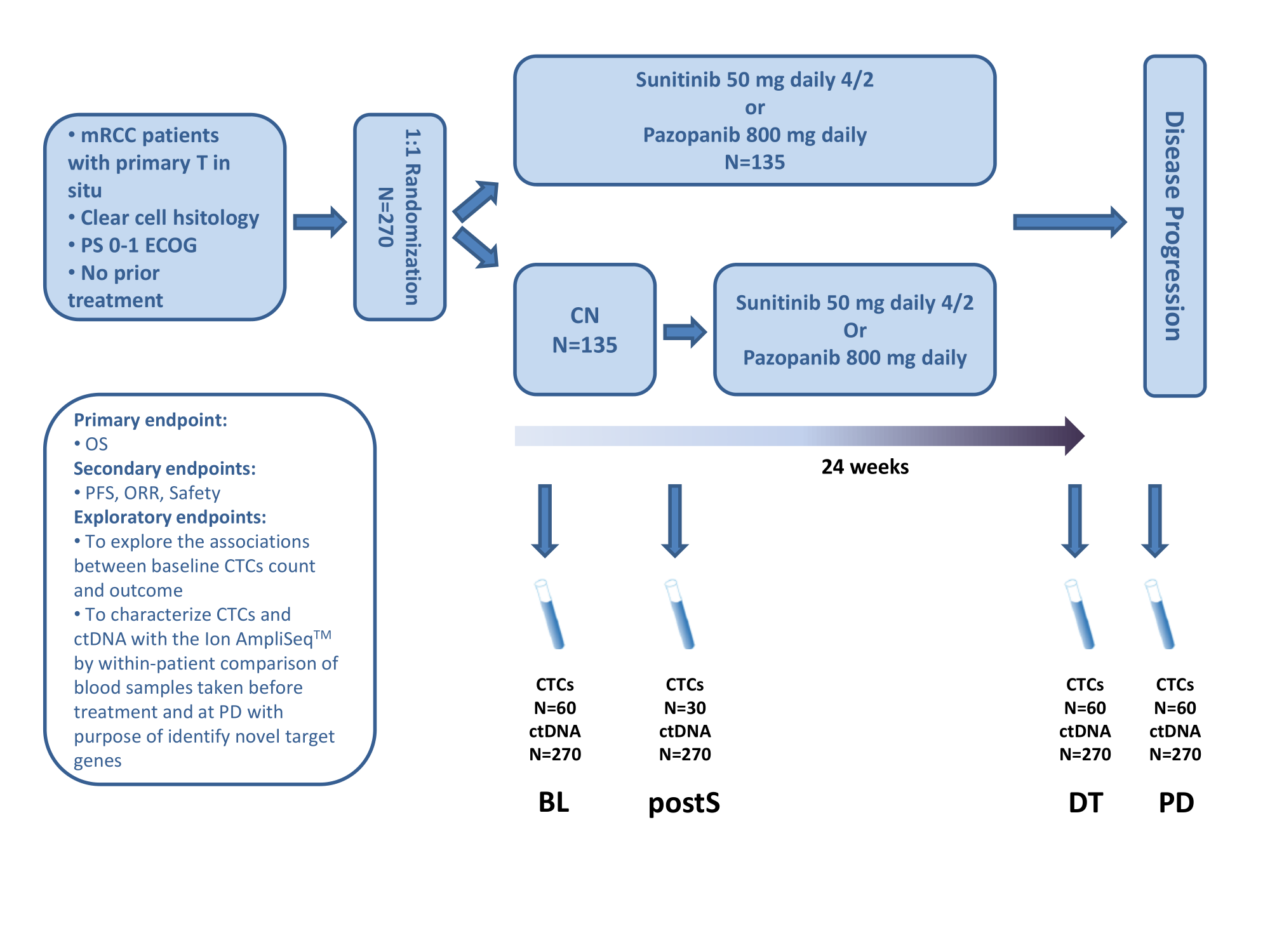

Supplement: Supplementary file 1 [file ijms-21-01475-s001.zip › SFig1.tif]

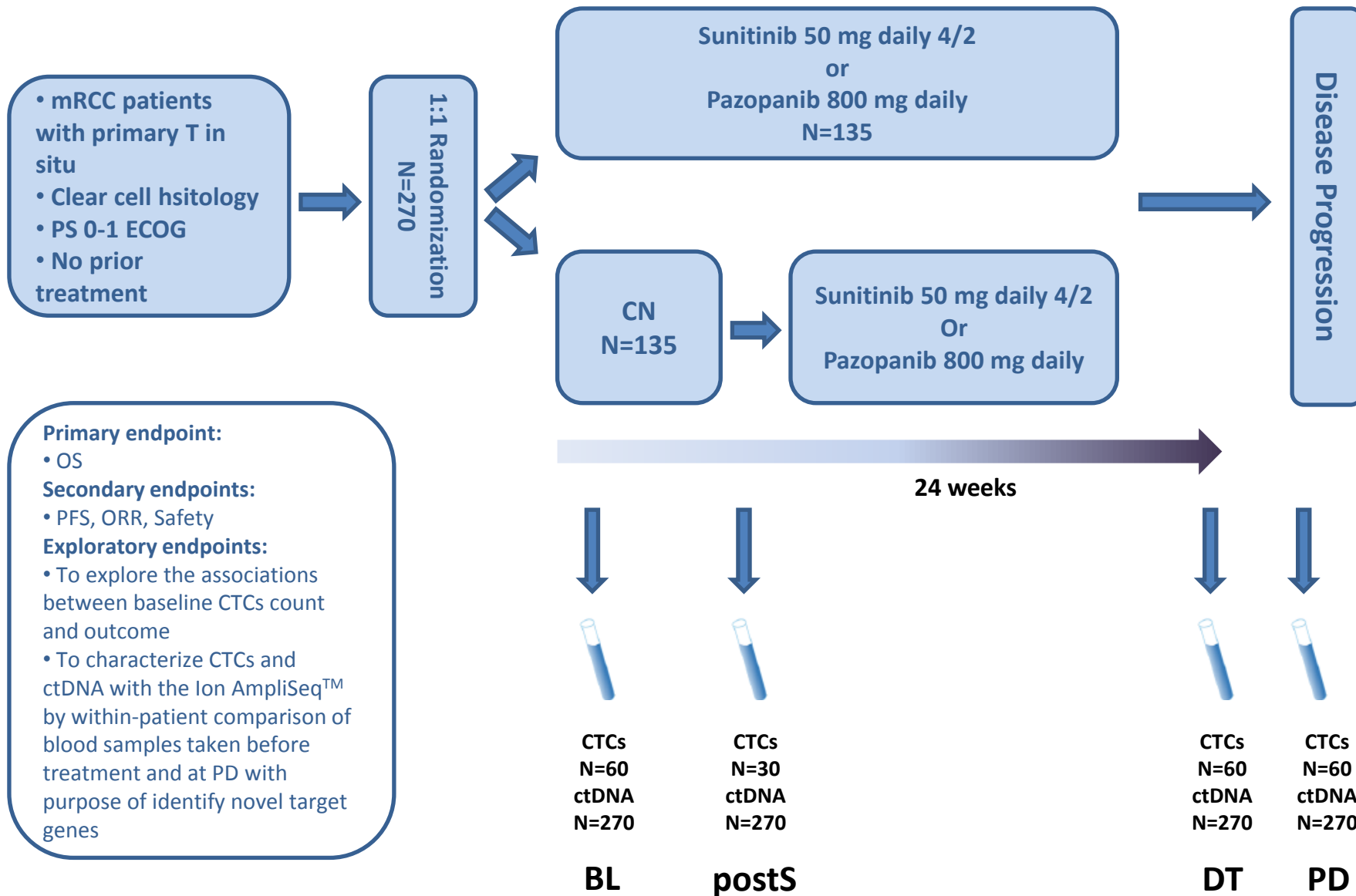

## OLD

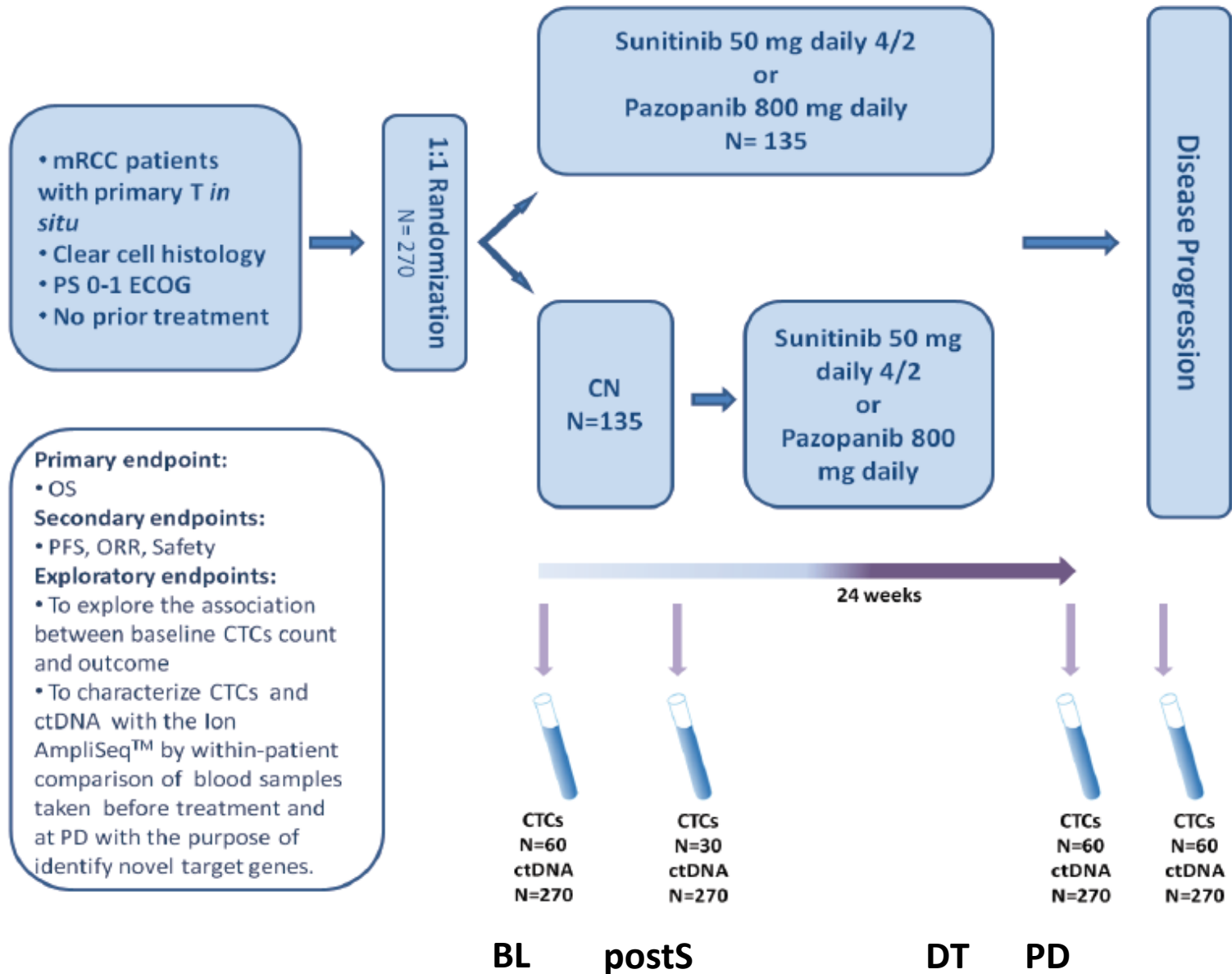

Supplement: Supplementary file 1 [file ijms-21-01475-s001.zip › SFig1.pdf]
